# Rapid antifungal phenotypic detection of *Candida tropicalis* based on laser-scattering technology combined with machine learning

**DOI:** 10.3389/fcimb.2026.1764421

**Published:** 2026-08-21

**Authors:** Jingfang Sun, Xinyi Liang, Shuang Song, Shulong Zhao, Xiaojie Zhao, Xinyi Deng, Jiaxin Li, Fei Jiang, Haiquan Kang

**Affiliations:** 1 Department of Laboratory Medicine, The Affiliated Hospital of Xuzhou Medical University, Xuzhou, China; 2 College of Medical Technology, Xuzhou Medical University, Xuzhou, China

**Keywords:** aloze resistant, antifungal susceptibility prediction, *Candida tropicalis*, laser-scattering signal, machine learning

## Abstract

**Background:**

Invasive fungal infections have become a significant cause of mortality among immunocompromised and critically patients. *Candida tropicalis* (*C. tropicalis*) has exhibited a marked increase inprevalence and a high rate of azole resistance as one of the major non *C. albicans* pathogens associated with bloodstream and deep infections. Therefore, the necessity for rapid and accurate susceptibility testing is paramount for the precise administration of treatment. The objective of this study was to develop a rapid prediction method for antifungal susceptibility based on early phenotypic signals (1–8 h) acquired through laser-scattering rapid culture technology.

**Methods:**

Antifungal susceptibility testing was performed on 85 clinical isolates of *C. tropicalis* using Sensititre Yeast one YO10 panels to determine the minimum inhibitory concentrations (MICs). The assay for the detection of antifungal susceptibility for *C. tropicalis* clinical isolates was designed with laser-scattering technology. The model input features comprised laser-scattering intensity values (scattering units) and growth-curve parameters (slope, growth rate, maximal scattering intensity, area under the curve, etc.) collected from 1–8 h of drug exposure. Machine-learning models targeting fluconazole (FLZ), voriconazole (VOR) were constructed using random forest (RF), support vector machine (SVM), logistic regression (Logit), and stacking ensemble algorithms. The performance of the model was evaluated through five-fold cross-validation, with the area under the curve (AUC), sensitivity, specificity, accuracy, and F1-score being used as the evaluation metrics.

**Results:**

The proportion of isolates demonstrating resistance to FLZ was 28.2% (24/85), while to VOR was 21.2% (18/85). With regard to the antifungal susceptibility prediction models, it was found that all machine-learning models achieved effective prediction within 6–8 hours of drug exposure. The FLZ and VOR models demonstrated the highest discriminative ability, with the Logit model for FLZ attaining an AUC of 0.923 and the SVM model for VOR achieving an AUC of 0.900. Notably, both models exhibited F1-scores that surpassed 0.88. Feature-importance analysis revealed that phenotypic signals from 5–8 h contributed most to prediction accuracy, reflecting the time-dependent nature of antifungal effects.

**Conclusions:**

Machine-learning models based on early phenotypic data have been shown to be capable of accurately predicting antifungal susceptibility within 8 hours.This represents a significant improvement over conventional methods and provides a promising technical framework for the development of rapid antifungal susceptibility testing and precision anti-infective therapy.

## Introduction

The incidence of fungal infections has increased significantly among critically and immunocompromised patients, with the most precalent invasive infections caused by *Candida* specie (Arendrup and Patterson, 2017; Dong et al., 2026; Miao et al., 2026). In recent years, the widespread clinical use of antifungal agents has been accompanied by a growing prevalence of resistance among clinical isolates to fluconazole (FLZ) and voriconazole (VOR) (Pfaller et al., 2019; Wang et al., 2021; Wan et al., 2023). A multicentre study showed that the resistance rates of *C. tropical* to fluconazole increased from 5.7% to 31.8%, and to voriconazole increased from 5.7% to 29.1% (Wang et al., 2021). This predicament presents substantial challenges to clinical treatment, complicates public health containment efforts, and creates significant obstacles in patient management. Conventional antifungal susceptibility testing (AFST) methods generally necessitate a duration of 24–72 hours to generate results. Empirical antifungal therapy is frequently initiated during the early phase of infection, which may result in a delay in the initiation of optimal treatment and the subsequent propagation of resistant strains. Therefore, developing a rapid and reliable phenotypic method that provides antifungal susceptibility information within a few hours is of critical importance for precision therapy and antimicrobial stewardship.

Laser light scattering-based rapid culture technology has recently offered a novel approach for microbiological diagnostics. This technique is characterised by its ability to monitor alterations in light-scattering intensity during the process of microbial growth. Then it employs a data-fitting process, whereby the collected data is fitted to nonlinear least-squares growth curves and enables a quantitative assessment of microbial growth dynamics (Bugrysheva et al., 2016; Idelevich et al., 2017; Wei et al., 2021; Yu et al., 2023; Huang et al., 2025). Compared with conventional growth-based methods, laser scattering approaches offer several distinct advantages: label-free, real-time and rapid (Kim et al., 2025; Yang et al., 2025). A study was conducted that utilised machine learning-assisted classification of pathogenic yeasts, achieving a high level of accuracy in yeast species identification through the analysis of laser light scattering patterns, with a success rate of 95.3% (Liu et al., 2026). But, the vast majority of laser scattering-based AFST studies have focused on bacterial pathogens (Wei et al., 2021; Kim et al., 2025). Systematic evaluations of laser scattering for fungal susceptibility testing, especially using clinically relevant *Candida* isolates, remain scarce. A paucity of research has been conducted on the performance of laser scattering technology for antifungal susceptibility testing of *C. tropicalis*, which has a high rate of azole resistance.

In this study, the laser-scattering rapid culture system was employed to collect dynamic phenotypic data from *C. tropicalis* exposed to fluconazole, voriconazole, and caspofungin for 1–8 hours. The objective of this study was to elucidate the quantitative relationship between early phenotypic signals and antifungal responses and to explore the potential of laser-scattering dynamics in rapid antifungal susceptibility testing.

## Materials and methods

### Strains

Between July 2023 and November 2024, 85 non-duplicated *C. tropicalis* clinical isolates were collected from the Department of Laboratory Medicine, the Affiliated Hospital of Xuzhou Medical University. For quality control, *Candida parapsilosis* ATCC 22019 and *Pichia kudriavzevii* ATCC 6258 were used for AFST, while *C. tropicalis* ATCC 750, Which has a clear genetic background, served as the reference strain for Laser-Scattering Technology assays. This study was approved by the Ethics Committee of the Affiliated Hospital of Xuzhou Medical University (Approval No.: XYFY2024-KL178-01).

### Strain identification and antifungal susceptibility testing

All clinical isolates were identified to the species level using a MALDI-TOF MS system (Zybio, Chongqing, China). Antifungal susceptibility was performed using the Sensititre YeastOne YO10 antifungal susceptibility plates (Thermo Scientific, USA) to determine the minimum inhibitory concentrations (MICs) of antifungal agents. MICs were read on color change according to the manufacturer’s instructions. Interpretive criteria followed the 2022 CLSI guidelines M27M44S-Ed3 (CLSI, 2022). According to the guidelines, isolates were classified as susceptible (S), intermediate (I), susceptible-dose dependent (SDD), resistant (R) based on their MICs.

### Laser-scattering technology assays for AFST

Laser-Scattering Technology assays was designed to detect susceptible or not for *C. tropicalis* clinical isolates used by HB&L culture system. Briefly, a HB&L bottle (Alifax, Italy) containing 2 mL broth and a magnetic stir bar, was inoculated with 140 µL of fungal suspension, and 10 µL antifungal solution—fluconazole (FLZ), voriconazole (VOR), or caspofungin (CAS)—at a final concentration of 2 µg/mL, 0.12 μg/mL and 0.25 μg/mL according to the susceptible breakpoints of 2022 CLSI guidelines M27M44S-Ed3, respectively. Cultures were incubated at 37 °C for 24 h, and the HB&L system automatically recorded scattering signals over time.

### Machine-learning model construction and performance evaluation

Early laser-scattering phenotypic data recorded by the HB&L system at 1**–**8 h (LS_1h–LS_8h) were used as quantitative model inputs. A total of eight temporal features were extracted and standardized using Z-score normalization. Four algorithms were implemented: random forest (RF), support vector machine (SVM), logistic regression (Logit), and stacking ensemble (Stacking). The SVM used a radial basis function (RBF) kernel with parameters optimized via grid search and five-fold cross-validation, followed by Platt scaling for probability calibration. The RF model employed 100**–**500 trees with the Gini index as the split criterion. The logistic regression model used L2 regularization to prevent overfitting. Stacking integrated RF, SVM, and Logit as base learners, with logistic regression serving as the meta-learner for final prediction.

Model performance was evaluated based on several key metrics, including accuracy, sensitivity, specificity, precision, the F1-score, and the area under the receiver-operating characteristic curve (AUC). To preserve the balance between susceptible and resistant samples, stratified five-fold cross-validation was employed. Mean values across folds were used to represent overall model performance and stability. Receiver-operating characteristic (ROC) and precision-recall (P–R) curves were plotted to evaluate discriminative ability and minority-class detection, respectively. Calibration curves and confusion matrices were analyzed to assess probability, reliability and misclassification patterns. A multidimensional evaluation framework was applied to compare algorithmic discrimination and generalization performance across all models.

### Data processing and statistical analysis

All data preprocessing, modeling, and visualization were performed in R (version 4.5.0) using RStudio (Posit, version 2025.09.0) as the integrated development environment.

## Results

### Antifungal susceptibility of clinical *C. tropicalis* isolates

A total of 85 non-duplicate *C. tropicalis* clinical isolates were included in this study, and the antifungal susceptibility results are summarized in **Table 1**. Furthermore, 54.1% (46/85) of the isolates were susceptible to FLZ, while 28.2% (24/85) isolates were resistant. For VOR, 57.6% (49/85) of isolates were susceptible, while 21.2% (12/85) isolates were resistant. Regarding echinocandins, CAS demonstrated excellent *in vitro* activity, with 82 isolates (96.4%) classified as susceptible and a mere 3 as intermediate. The results indicate a relatively higher resistance rate to azole drugs, whereas echinocandins remain highly activity against *C. tropicalis*.

**Table 1:** Antifungal susceptibility profiles of clinical *C. tropicalis* isolates (n = 85).

Antifungal agent	MIC50	MIC90	S	SDD/I	R
	(μg/mL)	(μg/mL)	n (%)	n (%)	n (%)
Fluconazole (FLZ)	2	256	46 (54.1)	15 (17.7)	24(28.2)
Voriconazole (VOR)	0.12	8	49 (57.6)	18 (21.2)	18(21.2)
Caspofungin (CAS)	0.12	0.25	82 (96.5)	3 (3.5)	0(0)

FLZ, fluconazole; VOR, voriconazole; CAS, caspofungin; S, susceptible; I, intermediate; SDD, susceptible-dose dependent; R, resistant.

### Antifungal susceptibility results from the laser-scattering culture system

**Figure 1:**
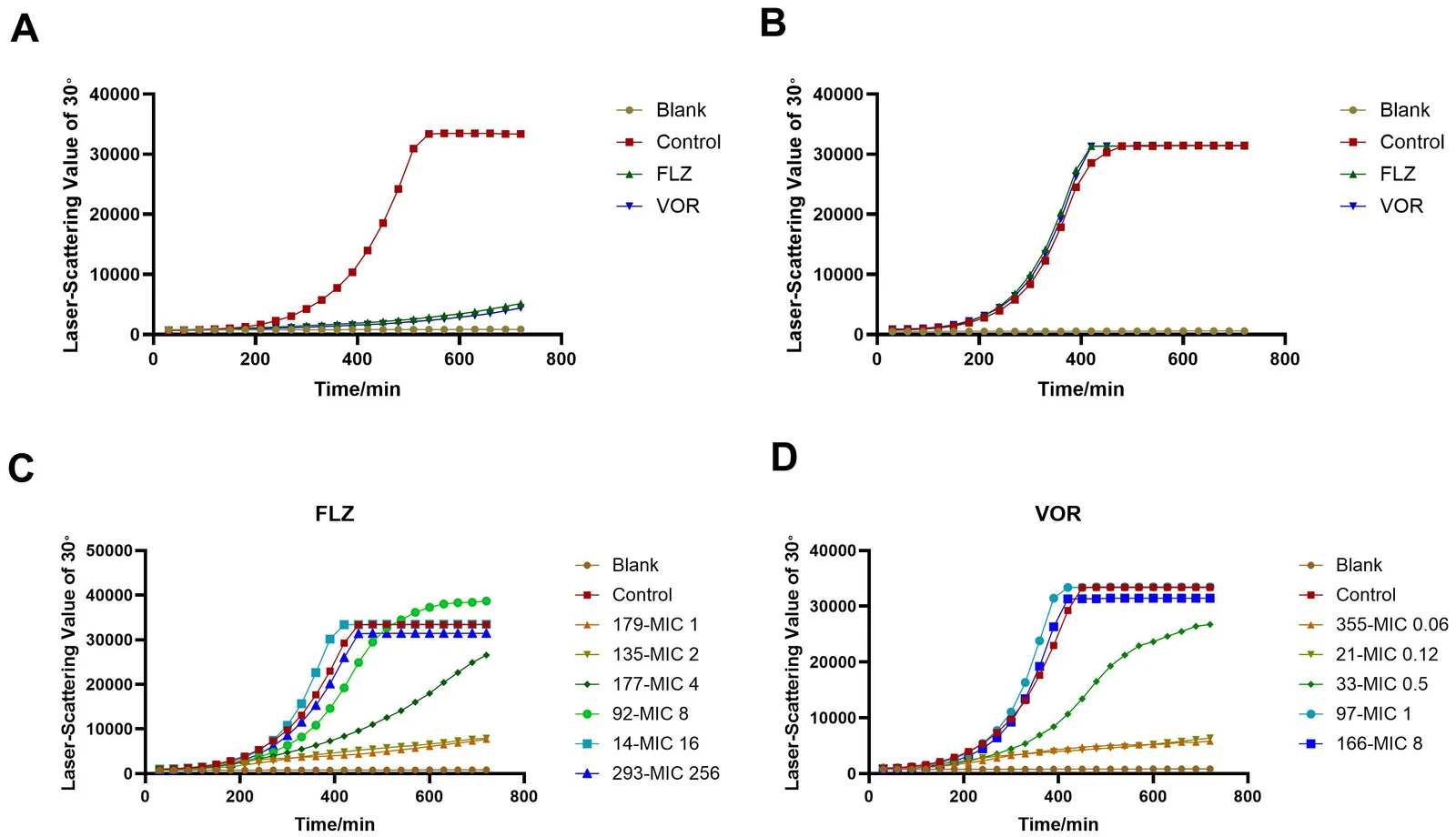
Growth curves of *C. tropicalis* obtained by the HB&L microbial culture system. **(A)** Growth curves of susceptible isolates; **(B)** Growth curves of resistant isolates; **(C)** Growth curves of different clinical strains under FLZ; **(D)** Growth curves of different clinical strains under VOR.

A total of 85 C.*tropicalis* isolates were included in this study. Laser-scattering Culture System can reflect the growth condition by monitoring changes of laser-scattering signal. The solvent, dimethyl sulfoxide (DMSO), has no effect on the growth of *C. tropicalis* (**Supplementary Figure 1A**). Based on the convenience of the operation and distinctiveness of results, we chose 0.5 McF as the inoculation concentration (**Supplementary Figures 1B, C**). As shown in **Figure 1**, it is possible to distinguish between sensitive strains and resistant strains through growth curves. These results indicate that laser-scattering technology assays can be used to monitor antifungal susceptibility of *C. tropicalis*. However, determining antifungal resistance directly from the growth curves can be subjective and potentially biased. Therefore, we further integrated the growth-curve data with machine-learning algorithms to construct predictive models and perform validation.

### Machine-learning models and performance evaluation

However, determining antifungal resistance directly from the growth curves may introduce subjectivity and potential bias. Therefore, we further integrated the growth-curve data with machine-learning algorithms to construct predictive models and perform validation. To further evaluate the discriminative capability of antifungal resistance phenotypes of *C. tropicalis* based on the laser-scattering culture system, four machine-learning models were constructed: RF, SVM, Logit and Stack. Due to the limited number of intermediate isolates and the resulting skewed data distribution, SDD/I categories were merged with R group, which was categorised as not susceptible(NS).

**Figure 2:**
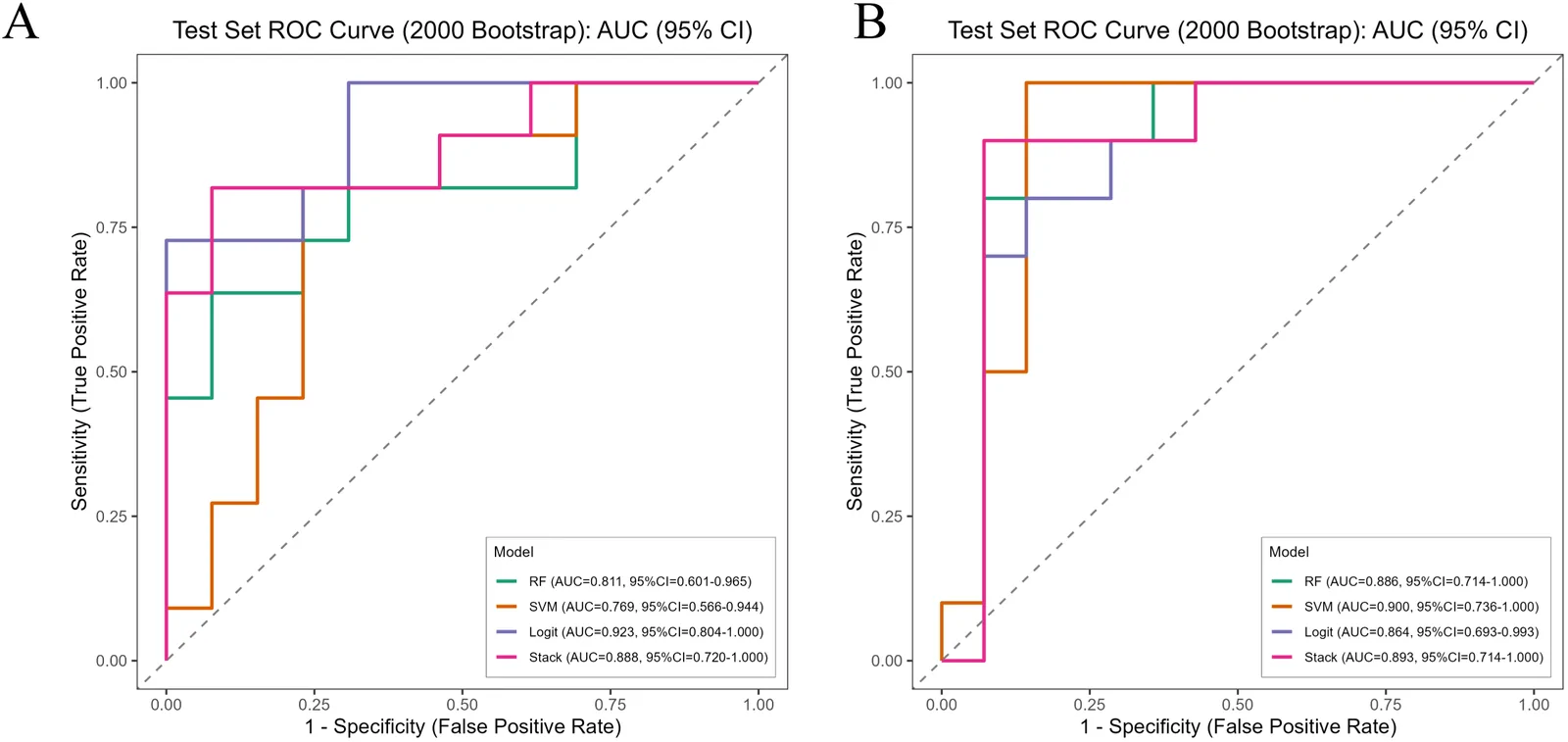
ROC curves of different machine-learning models for antifungal susceptibility prediction in *C. tropicalis*. **(A, B)** represent prediction results for FLZ and VOR, respectively. RF, random forest; SVM, support vector machine; Logit, logistic regression; Stack: and stacking ensemble.

This adjustment aimed to improve the stability and generalisability of the model for predicting clinical susceptibility. **Figure 2** shows the ROC curves and AUC performance of the four models for predicting antifungal susceptibility to FLZ and VOR. Overall, all models demonstrated satisfactory discriminative power on the test set, with AUC values exceeding 0.75. The Logit for FLZ achieved the best overall performance, with an AUC of 0.923 (95% CI: 0.804–1.000). For VOR, the SVM model yielded an AUC of 0.900 (95% CI: 0.736–1.000), indicating superior generalization ability. These findings imply that machine-learning models, when trained using early phenotypic data within 8 hours from the HB&L system, can accurately distinguish between NS and S *C. tropicalis* isolates. Among the tested algorithms, the SVM model exhibited the best discriminative performance and robustness.

**Figure 3:**
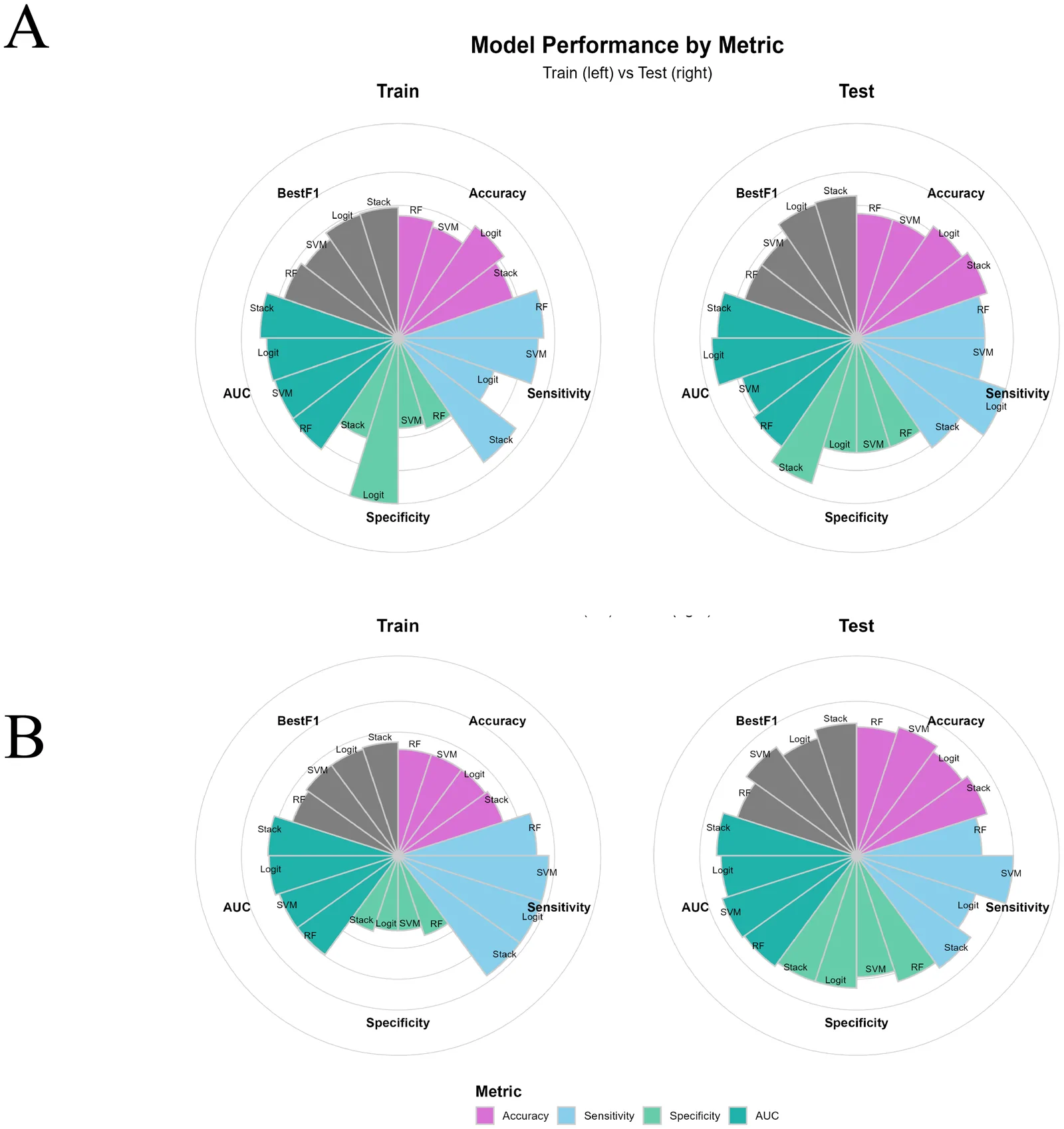
Comprehensive performance radar plots of different machine-learning models for antifungal susceptibility prediction in *C. tropicalis*. **(A, B)** represent the comprehensive performance of the FLZ and VOR prediction models in the training set (left) and test set (right), respectively.

**Figure 3** shows the overall performance of the four machine-learning models in predicting antifungal susceptibility to FLZ and VOR. The models exhibited stable performance between the training and test sets, with no evidence of overfitting. The Logit algorithm demonstrated the best comprehensive performance on the test set for FLZ model, achieving an AUC of 88.81%, F1-score of 85.71%, specificity of 92.31%, 0.746 of Kappa coefficient and an overall accuracy of 87.50%. For the VOR models, all performance metrics were high, with the SVM model achieving an AUC of 90.00%, F1-score of 86.96%, sensitivity of 100.00%, 0.753 of Kappa coefficient, and accuracy of 87.50%, indicating strong predictive capability. Radar plots of the five key performance metrics—accuracy, sensitivity, specificity, AUC, and F1-score—revealed that the stacking ensemble model achieved a better balance between sensitivity and specificity in FLZ prediction, while the SVM model performed best in VOR susceptibility prediction.

Taken together, these findings suggest that machine-learning models trained using early phenotypic data from the HB&L system are highly stable and reproducible in terms of their ability to predict antifungal susceptibility. The stacking ensemble strategy and SVM algorithm exhibited the most robust and consistent performance, providing methodological support for rapid, intelligent interpretation of antifungal susceptibility.

**Figure 4:**
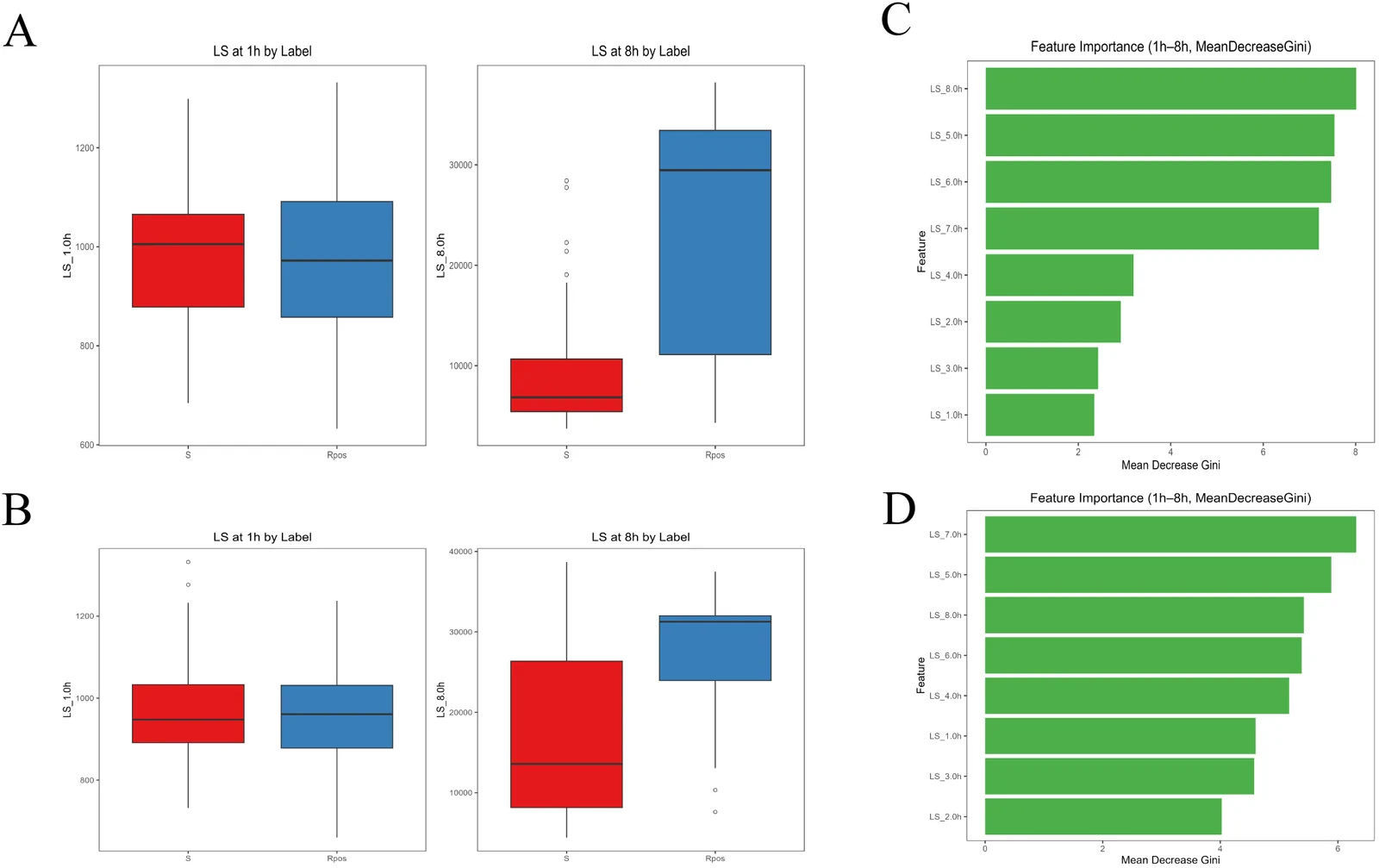
Key phenotypic feature distribution and importance analysis of machine-learning models for different antifungal agents. **(A, B)** show the distribution differences of the 1-hour (LS_1.0h) and 8-hour (LS_8.0h) phenotypic signals between susceptible (S) and not susceptible(NS) isolates in the FLZ and VOR models, respectively. **(C, D)** present the feature importance ranking across time points (1–8 h) in the optimal models, calculated based on the mean decrease in Gini index. Higher values indicate greater contribution of the corresponding feature to model discrimination.

Following confirming the overall stability of the machine-learning models for predicting antifungal susceptibility, a further analysis of feature-level interpretability was performed (**Figure 4**). The results indicated significant variations in early (1 h) and late (8 h) phenotypic signal distributions among the models for various antifungal agents. In the FLZ Logit model and the VOR SVM model, NS isolates exhibited significantly higher laser-scattering (LS) values at 8 hours compared with S isolates (S) (**Figures 4A, B**). This pattern suggests that NS strains exhibited stronger growth activity under antifungal exposure, whereas susceptible strains demonstrated evident growth inhibition, indicating that the phenotypic effects of antifungal agents are time dependent. Feature importance analysis (**Figures 4C, D**) further demonstrated that, in both the FLZ Logit and VOR SVM models, variables corresponding to LS signals from 5.0 h to 8.0 h had the highest mean decrease in Gini index, contributing substantially more than early (1–3 h) signals. These findings indicate that the models primarily rely on mid- to late-phase growth features to discriminate between resistant and susceptible phenotypes.

Collectively, these results suggest that the antifungal response of *C. tropicalis* exhibits strong time dependence, with phenotypic signals recorded between 5–8 hours serving as key indicators for distinguishing resistant from susceptible isolates.

## Discussion

Invasive Candida infections have become a significant concern for immunocompromised and critically ill patients, with the isolation rate of *C. tropicalis* steadily increasing across the Asia–Pacific region and showing a high prevalence of azole resistance (Pfaller et al., 2019; Wang et al., 2021; Wan et al., 2023). However, conventional antifungal susceptibility testing methods require 24–72 hours to generate results, limiting their usefulness in guiding early antifungal therapy. The advent of rapid phenotypic analysis technologies has precipitated the emergence of a promising approach that utilises microbial growth dynamics for the purpose of predicting susceptibility (Bugrysheva et al., 2016; Idelevich et al., 2017; Idelevich et al., 2018; Wei et al., 2021; Yi et al., 2023; Yu et al., 2023; Falconer et al., 2024). For instance, the utilisation of laser scattering technology has been demonstrated to reduce the time required to determine antimicrobial susceptibility by between 50% and 75% for *Bacillus anthracis*, *Yersinia pestis* and *Burkholderia pseudomallei*. The process was completed within a timeframe of six hours, allowing for the distinction between resistant and susceptible cases (Bugrysheva et al., 2016). Subsequent investigations revealed that changes in light-scattering curves induced by antimicrobialscan reliably reflect growth inhibition patterns, thus enabling rapid testing of antimicrobial susceptibility phenotypes.

For carbapenem-resistant Enterobacterales, the light-scattering curves demonstrated a high degree of accuracy, exhibiting 100% sensitivity and 96.0% specificity at a mere 6 hours of culture time (Wei et al., 2021). A study of HB&L system for the rapid diagnosis of UTIs compared with traditional urine culture methods showed that laser scattering technology can quickly detect fungal infections in urine samples, such as *Candida glabrata*, *C. tropicalis*, et al. in urine samples (Huang et al., 2025), suggesting that light-scattering signal could monitor growth of *Candida* species. Collectively, these findings emphasise the substantial capability of laser-scattering systems to capture real-time phenotypic responses, providing a robust foundation for rapid antifungal susceptibility prediction in *C. tropicalis*.

Laser scattering technology has emerged as a novel approach for the direction of drug resistance detection, a development that mirrors the prevailing trend of microbial growth. Nevertheless, the utilisation of the growth curve as the sole criterion for determining strain resistance is susceptible to subjective errors in judgment. Machine learning models have been widely applied in identify species and drug resistance among bacteria and fungi (Stöckel et al., 2017; Gu et al., 2022; Falconer et al., 2024; Kang et al., 2024). In this study, machine-learning models were developed based on early (1–8 h) phenotypic laser-scattering data obtained from the HB&L culture system to achieve rapid antifungal susceptibility prediction for *C. tropicalis*. The models for FLZ and VOR achieved AUC values of 0.923 and 0.900, respectively, both exceeding the empirical benchmark of 0.85, indicating strong discriminative power. Although the CAS model showed a slightly lower AUC (0.875) due to class imbalance, it maintained stable predictive capability (data was not shown). It is important to note that the analysis of model interpretability revealed that phenotypic signals between 5–8 hours contributed most to resistance discrimination, indicating a clear time-dependent drug response pattern. The superior performance of the Logit and SVM models for FLZ and VOR, respectively, further suggests that algorithmic complementarity and ensemble learning can enhance model robustness and generalization. In this study, stability of the model was validated through 2, 000 bootstrap iterations, thereby confirming that machine learning–based analyses can yield reliable and reproducible results even with relatively small sample sizes.

Moreover, the HB&L laser-scattering system utilised in this study facilitated the acquisition of growth curves in real-time with enhanced sensitivity and quantitative precision compared to conventional optical density or colony-counting methods. Previous studies have reported its excellent accuracy and versatility in rapid bacterial culture for bloodstream and urinary tract infections (Athamna and Freimann, 2019; Falconer et al., 2024; Huang et al., 2025). This study applied the HB&L system to fungal susceptibility modelling combined with machine learning. The findings demonstrate that early scattering signals can reflect fungal tolerance dynamics under antifungal exposure, providing a novel framework for rapid (≤8 h) antifungal susceptibility testing and paving the way toward an integrated “culture–identification–prediction” workflow in clinical microbiology laboratories.

Nevertheless, several limitations should be acknowledged. The sample size was relatively small and derived from a single-center study, thus necessitating multi-centre validation to enhance external generalisability. Furthermore, a head-to-head comparison between the new method and the CLSI and/or EUCAST reference broth microdilution methods is planned, using a larger collection of clinical isolates. The CAS dataset contained a limited number of resistant isolates, which may have resulted in biased classification thresholds. Future research should address this through balanced sampling, feature augmentation, and inclusion of independent validation cohorts. In addition, the absence of formal intra- and inter-assay CV data is a limitation of this preliminary study; future multicenter studies with large cohorts are warranted to rigorously validate the repeatability of this laser-scattering platform. This study established a preliminary evaluation model to distinguish susceptible or non-susceptible strains of *C. tropicalis* by using laser scattering combined with machine learning. Nevertheless, the results of this system were still based on pure culture. The model had the potential to facilitate the detection drug susceptibility in positive blood cultures and in conjunction with the establishment of a calibration curve to quantitatively correlate laser scattering signal intensity with MICs values, thereby enabling the reporting of specific MICs values. It is evident that there is an area that requires further development and exploration.

## Conclusion

The machine-learning models developed in this study, leveraging early phenotypic features captured by the laser-scattering rapid culture system, demonstrated high discriminatory power in predicting *C. tropicalis* antifungal susceptibility profiles within 8 hours, markedly shortening the conventional turnaround time. These findings highlight the time-dependent nature of fungal drug responses and provide a novel methodological basis for rapid antifungal susceptibility testing, contributing valuable insights for intelligent resistance surveillance and individualized antifungal therapy.

## Data Availability

The raw data supporting the conclusions of this article will be made available by the authors, without undue reservation.
